# Intervention with inulin prior to and during sanative therapy to further support periodontal health: study protocol for a randomized controlled trial

**DOI:** 10.1186/s13063-021-05504-1

**Published:** 2021-08-10

**Authors:** Carly A. R. Zanatta, Peter C. Fritz, Elena M. Comelli, Wendy E. Ward

**Affiliations:** 1 Department of Kinesiology, Faculty of Applied Health Sciences, Brock University, St. Catharines, ON Canada; 2 Center for Bone and Muscle Health, Faculty of Applied Health Sciences, Brock University, St. Catharines, ON Canada; 3 Periodontal Wellness & Implant Surgery, Fonthill, ON Canada; 4 Department of Nutritional Sciences and Joannah and Brian Lawson Centre for Child Nutrition, Faculty of Medicine, University of Toronto, Toronto, ON Canada

**Keywords:** Inulin, Periodontal disease, Prebiotics, Sanative therapy, Gut microbiome

## Abstract

**Background:**

Periodontal disease is a chronic state of inflammation that can destroy the supporting tissues around the teeth, leading to the resorption of alveolar bone. The initial strategy for treating periodontal disease is non-surgical sanative therapy (ST). Periodontal disease can also induce dysbiosis in the gut microbiota and contribute to low-grade systemic inflammation. Prebiotic fibers such as inulin can selectively alter the intestinal microbiota and support homeostasis by improving gut barrier functions and preventing inflammation. Providing an inulin supplement prior to and post-ST may influence periodontal health while providing insight into the complex relationship between periodontal disease and the gut microbiota. The primary objective is to determine if inulin is more effective than the placebo at improving clinical periodontal outcomes including probing depth (PD) and bleeding on probing (BOP). Secondary objectives include determining the effects of inulin supplementation pre- and post-ST on salivary markers of inflammation and periodontal-associated pathogens, as these outcomes reflect more rapid changes that can occur.

**Methods:**

We will employ a single-center, randomized, double-blind, placebo-controlled study design and recruit and randomize 170 participants who are receiving ST to manage the periodontal disease to the intervention (inulin) or placebo (maltodextrin) group. A pilot study will be embedded within the randomized controlled trial using the first 48 participants to test the feasibility for the larger, powered trial. The intervention period will begin 4 weeks before ST through to their follow-up appointment at 10 weeks post-ST. Clinical outcomes of periodontal disease including the number of sites with PD ≥ 4 mm and the presence of BOP will be measured at baseline and post-ST. Salivary markers of inflammation, periodontal-associated pathogens, body mass index, and diet will be measured at baseline, pre-ST (after 4 weeks of intervention), and post-ST (after 14 weeks of intervention).

**Discussion:**

We expect that inulin will enhance the positive effect of ST on the management of periodontal disease. The results of the study will provide guidance regarding the use of prebiotics prior to and as a supportive adjunct to ST for periodontal health.

**Trial registration:**

ClinicalTrials.gov NCT04670133. Registered on 17 December 2020.

## Administrative information

**Table:** 

**Title {1}**	Intervention with inulin prior to and during sanative therapy to further support periodontal health: study protocol for a randomized controlled trial
**Trial registration {2a and 2b}.**	The study and its outcomes were registered on Clinical Trials.gov on December 17, 2020
**Protocol version {3}**	Version 2 of 17-12-2020
**Funding {4}**	To date, this research has not received a specific grant from any funding agency and if without external funding will be funded internally.
**Author details {5a}**	^1^Department of Kinesiology, Faculty of Applied Health Sciences, Brock University, St. Catharines, ON, Canada ^2^Center for Bone and Muscle Health, Faculty of Applied Health Sciences, Brock University, St. Catharines, ON, Canada ^3^Periodontal Wellness & Implant Surgery, Fonthill, ON, Canada ^4^Department of Nutritional Sciences and Joannah and Brian Lawson Centre for Child Nutrition, Faculty of Medicine, University of Toronto, Toronto, ON, Canada
**Name and contact information for the trial sponsor {5b}**	Not applicable
**Role of sponsor {5c}**	Not applicable

## Introduction

### Background and rationale {6a}

Periodontal disease is a non-reversible chronic inflammatory disease that is characterized by tissue damage of the supporting structures of teeth including alveolar bone loss. If untreated, tooth loss may result. Key determinants to the initiation and progression include genetic factors, tooth/dentition-related factors, comorbidities (systemic diseases), microbial communities (dental biofilms), and modifiable lifestyle factors (diet and smoking status) [1]. A mainline strategy for treating periodontal disease is non-surgical sanative therapy (ST). This procedure mechanically removes biofilm and calculus that contains pathogenic bacteria using ultrasonic and hand instrumentation. ST is effective at reducing the probing depths in the majority of sites where periodontal disease is present [2, 3] though ST is limited in its ability to remove organisms and calculus in deep periodontal pockets (> 5 mm), furcation areas (bone loss at the base of the tooth where roots meet), as well as within the tissues lining the periodontal pocket [4, 5]. Thus, the underlying causes of inflammation may not be completely eliminated. The effectiveness of ST can be assessed by measuring the changes in clinical outcomes such as probing depth (PD), clinical attachment loss, bleeding on probing (BOP), gingival index, plaque index, furcation involvement, and tooth mobility. However, the new 2017 classification of periodontal disease [6] needs to be taken into account when choosing a primary outcome of evaluating periodontal health. Classification for periodontal disease is now based on a multidimensional staging and grading system. PD is a critical clinical factor involved in determining the staging; the process of classifying the severity and complexity of the periodontal disease. It is also associated with healing after ST [3]. The absence of BOP can serve as a predictor of periodontal stability [7]. Together, both PD and BOP are clinically meaningful outcomes that can be used to determine a patient’s current periodontal status and their response to ST.

There is evidence of a link between our oral and gut microbiota, which is maintained in health and disease. Periodontal-associated pathogens, such as *Porphyromonas gingivalis*, *Prevotella intermedia*, and *Tannerella forsythia* have been found to range from 10^5^ to 10^8^ colony-forming units/mL of saliva in patients with periodontal disease [8, 9]. These bacteria are thought to travel through the proximal regions of the gastrointestinal tract and if they are able to survive the harsh conditions in the stomach, may eventually reach the large intestine [10]. Individuals with periodontal disease were shown to have present lower diversity of their gut microbiomes [11]. *P. gingivalis*, often considered the etiological agent of periodontitis, significantly alters the gut microbiota composition [12]. Changes in gut microbiota were associated with increased concentration of serum endotoxins, increased systemic inflammation, and decreased performances in both glucose and insulin tolerance tests [12]. In humans, the prevalence of severe periodontitis increases with increasing insulin resistance in nondiabetic individuals [13]. Chronic low-grade inflammation, defined as the increased production of pro-inflammatory cytokines and C-reactive protein (CRP), is a critical underlying factor that can contribute to an individual’s periodontal health [14]. A dysbiotic gut microbiota, present in obesity, diabetes, and metabolic syndrome, may be a contributor to this inflammation [15]. Thus, it is plausible that a dynamic loop exists between the oral and the gut microbiota which is altered in inflammatory states and modifiable by interventions targeting the oral and/or gut ecosystems. In summary, a strategy that targets gut bacteria, including prebiotics, may be useful in supporting periodontal health.

**Fig. 1:**
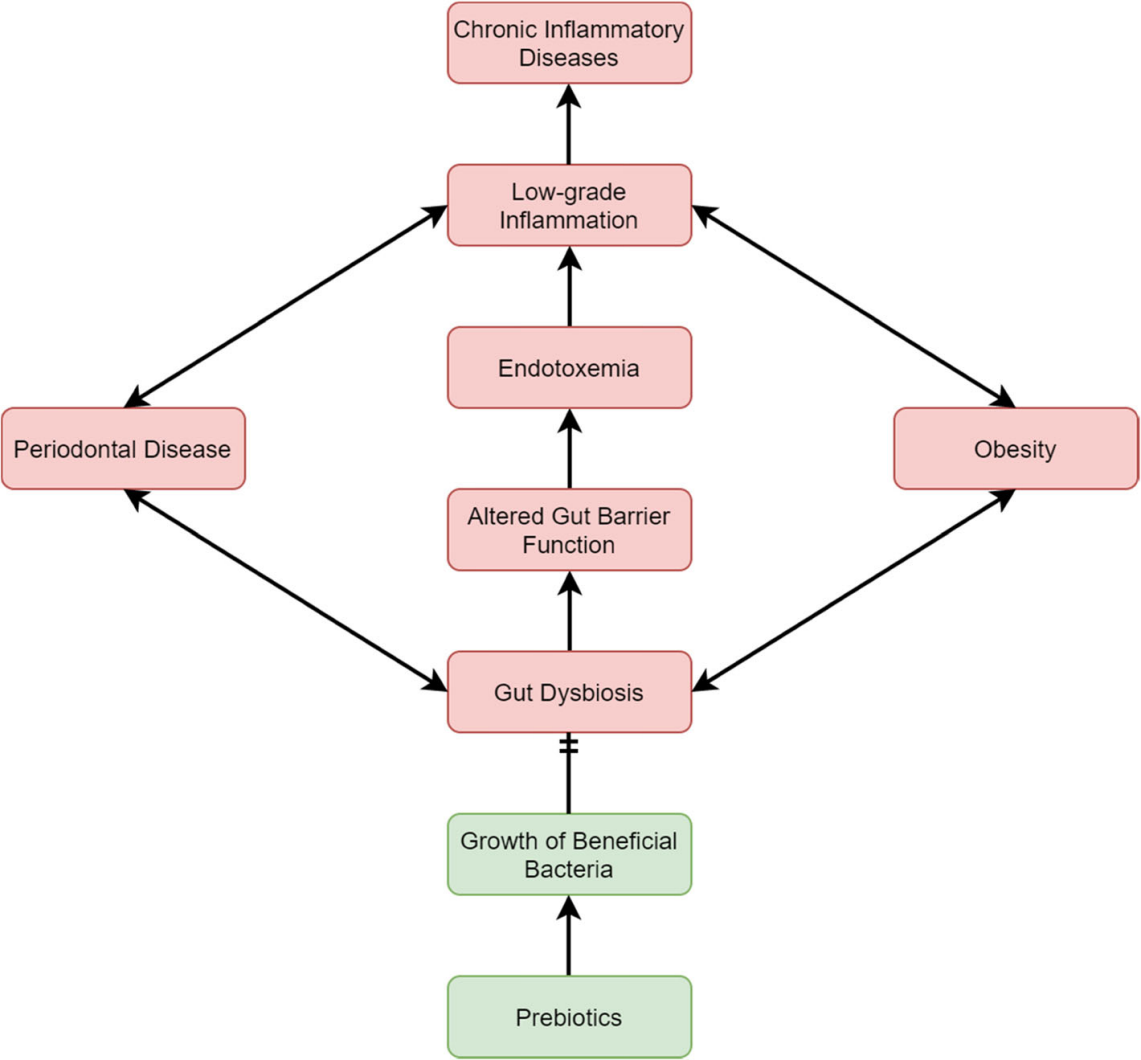
Possible connection linking periodontal disease, obesity, gut dysbiosis, and low-grade inflammation. Prebiotics may restore the gut microbiome to a state of homeostasis to disrupt the connection

Prebiotic administration may be a successful strategy in this context. Prebiotics are defined as substrates that are selectively utilized by host microorganisms conferring a health benefit [16]. Inulin-type fructans are among the most largely substantiated prebiotics. Inulin has a degree of fructose polymerization (DP) between 2 and 60, is undigested by the host, and selectively stimulates gut bacteria. Long-chain (DP = 10–60) inulin supplementation has been shown to lead to an increased production in butyrate, a short-chain fatty acid important in the maintenance of a healthy intestinal epithelium [17, 18]. Prebiotic supplementation and the production of butyrate may help to sustain the intestinal barrier leading to lower serum lipopolysaccharide (LPS) concentrations and improvement of low-grade chronic inflammation (Fig. 1) [19, 20].

While the effects of inulin supplementation on periodontal disease have not been investigated, epidemiological studies have reported a significant association between intake of dietary fiber and a decreased risk of periodontal disease [21–23]. Aligned with this, higher intakes of fruits and vegetables, including inulin-containing bananas, asparagus, and leeks, were significantly associated with a lower percentage of sites with PD greater than > 3 mm after ST [3]. One intervention study provided individuals with a low-fat, high fiber test meal three times a day for 8 weeks. At the end of the intervention, participants experienced improvements in periodontal disease outcomes that included significant decreases in average PD, clinical attachment levels, and percentage of sites with BOP [24]. Of note is that participants had a body mass index (BMI) of at least 25 kg/m^2^ or demonstrated impaired glucose tolerance—two important risk factors of developing periodontal disease. Although the mechanistic action could not be established in these studies, it is clear that intake of high-fiber foods has benefits for periodontal disease. The main aim of the study is to determine if supplementation with inulin will have beneficial effects on clinical outcomes of periodontal disease in patients with periodontal disease.

### Objectives {7}

The primary objective of this trial is to determine if inulin supplementation for 14 weeks, provided pre-, peri-, and post-ST, is more effective than the placebo at significantly decreasing the mean number of sites with PD ≥ 4 mm and decreasing the presence of BOP. Secondary objectives include determining the effects of inulin supplementation pre-, peri-, and post-ST on salivary markers of inflammation and periodontal-associated pathogens.

### Trial design {8}

**Fig. 2:**
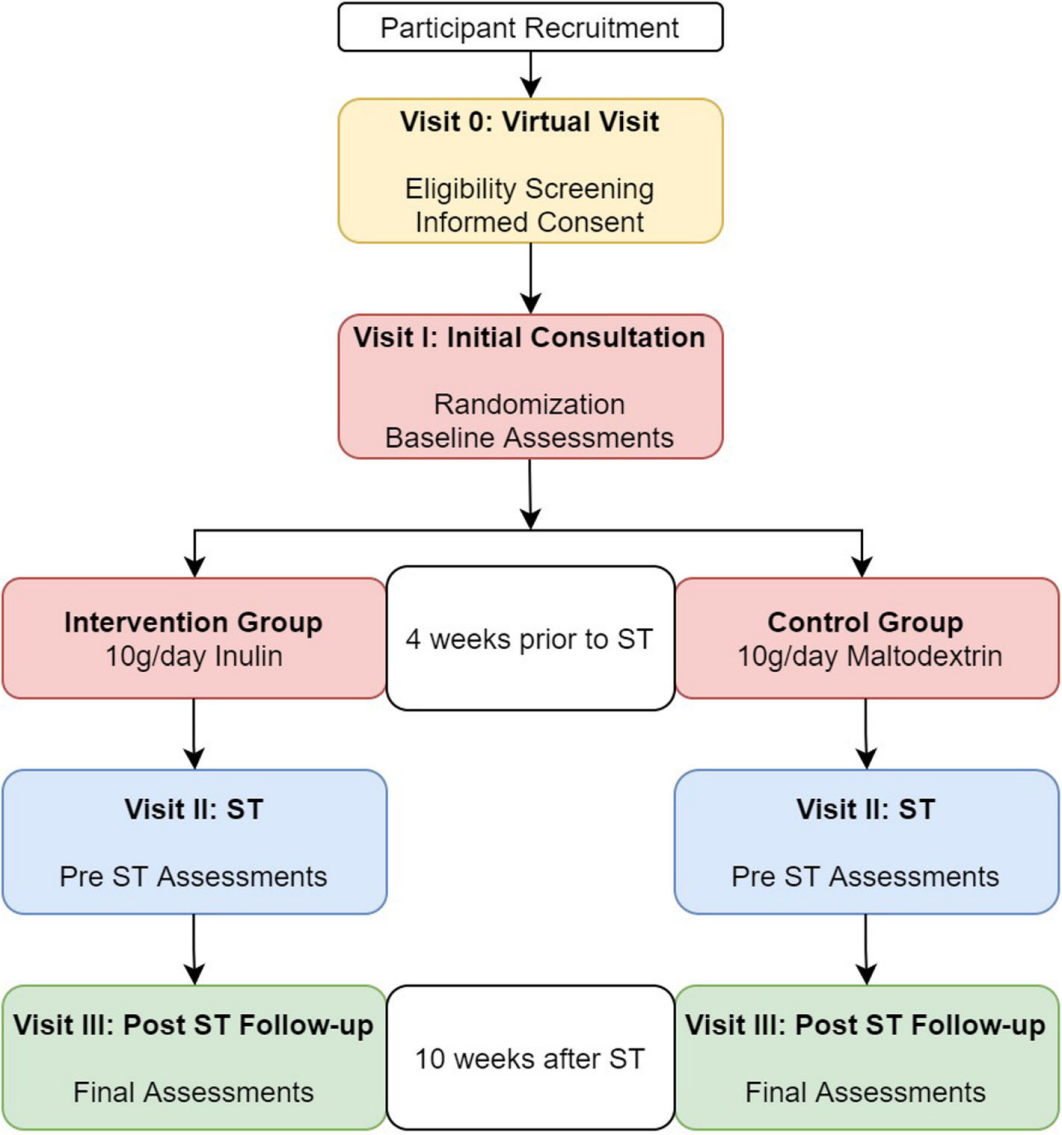
Study design flowchart

A pilot study will be embedded within the randomized controlled trial with the first 48 participants. Once the pilot study is completed and the feasibility has been established, these participants will be included in the full trial. This is a single-center, randomized, double-blind, placebo-controlled study. The flowchart of the study is presented in Fig. 2. Participants will be randomly allocated in a 1:1 ratio into the intervention group (*N* = 85) or control group (*N* = 85).

The visits and timeline is described below:

- Visit 0 (virtual visit): Eligibility screening and informed consent will follow if the participant meets the criteria.
- Visit I (initial consult): Informed consent (if not given virtually at Visit 0). Randomization to either inulin or placebo group. Baseline periodontal assessment (PD and BOP), anthropometry (weight, height), dietary assessment, salivary markers of inflammation, and periodontal-associated pathogens
- Visit II (ST is performed): Anthropometry (weight, height), dietary assessment, salivary markers of inflammation, and periodontal-associated pathogens. ST is typically scheduled 4 weeks from the patient’s initial consultation.
- Visit III (follow-up, post-ST): Final periodontal assessment (PD and BOP), anthropometry (weight, height), dietary assessment, salivary markers of inflammation, and periodontal-associated pathogens.

## Methods: Participants, interventions, and outcomes

### Study setting {9}

Participants will be recruited from a local periodontal clinic. Patients are referred to the specialty clinic by their general dentist or hygienist if they exhibit signs of advanced periodontal disease. Referred patients will be sent an email including the letter of invitation to participate in the study.

### Eligibility criteria {10}

Inclusion criteria include patients of both sexes, attending the clinic with a diagnosed periodontal disease requiring ST and over the age of 19 who are willing and able to provide informed consent.

Exclusion criteria included pregnancy, breast-feeding, demonstrating glycated hemoglobin (HbA1c) levels greater than 8% in the previous 3 months, chronic GI conditions (e.g., colon cancer, inflammatory bowel disease, celiac disease) and infections, use of antibiotics for the management of non-periodontal conditions within the previous 4 weeks, individuals who cannot stop taking laxatives, prebiotics, probiotics, and/or fiber supplements 2 weeks prior to the start of the intervention and for the duration of the study and current smoking and/or cannabis use. Patients with severe periodontal disease that require antibiotics with ST as part of their treatment will be withdrawn. This will be determined after the patients’ initial consultation (Visit I). Patients will also be withdrawn if they do not come back for Visit II or III. The eligibility criteria are summarized in Table 1.

**Table 1:** Participant inclusion, exclusion and withdrawal criteria

Inclusion criteria	Exclusion criteria	Withdrawal criteria
· Patients of both sexes attending the clinic with periodontal disease · Over the age 19 (no upper age restriction) · Be able to provide informed, written, or verbal consent	· Demonstrating HbA1c levels greater than 8% in the previous 3 months · Chronic GI conditions (e.g., colon cancer, inflammatory bowel disease, celiac disease) and infections · Current or previous use of antibiotics for management of non-periodontal conditions within the past 4 weeks · Individuals who cannot stop taking laxatives, prebiotics, probiotics and/or fiber supplements 2 weeks prior to the start of the intervention and for the duration of the study · Smokers and/or cannabis users · Pregnant or lactating	· Patients with severe periodontal disease that require antibiotics with ST as part of their treatment · Patients who do not show for Visit II or Visit III

### Who will take informed consent? {26a}

Those that are interested in participating in this study will be invited to a virtual meeting with a member of the research team due to the constraints of the COVID-19 pandemic. Participants can either agree to being virtually recorded as they read the statement from the consent form, fill out the consent form, and send it back via email or bring the consent form with them to their initial consultation (Visit I).

### Additional consent provisions for collection and use of participant data and biological specimens {26b}

A proportion of patients continue at the clinic for long-term/indefinite maintenance of their periodontal health. We may perform a follow-up of the participants after 1 or 2 years or later from the end of this study if they are continuing to have periodontal maintenance appointments at the clinic. If the participant wishes to be involved with the follow-up study, there is a line on the consent form that they can sign.

## Interventions

### Explanation for the choice of comparators {6b}

Participants allocated to the study group will receive inulin (10 g/day; Orafti®GR, Beneo, Mannheim, Germany) while participants in the placebo group will receive maltodextrin (10 g/day; Canadian Protein; Toronto, Ontario, Canada). Maltodextrin is the most common placebo used in intervention studies with inulin because it has a similar physical appearance and energy content to inulin.

### Intervention description {11a}

The supplements will be individually packaged in sachets and will contain 10 g of the specified powder. All participants will be instructed to consume one package per day. Supplements can be mixed with water or with another liquid of choice (250 mL of liquid per serving).

### Criteria for discontinuing or modifying allocated interventions {11b}

Inulin administered at 10 g/day is generally well-tolerated, resulting in only mild gastrointestinal (GI) symptoms of gas/bloating, flatulence, constipation, and GI cramping/rumbling [25]. Furthermore, participants will be instructed to divide the 10 g dose into two 5 g doses—in the morning and evening. This method has been shown to have no significant increase in GI symptom incidence [26]. We believe this will minimize, if any, side effects from the intervention. If the participant cannot continue with the study for any reason, they will be aware they may stop the intervention at any time and it will not affect the level of care they receive at the clinic.

### Strategies to improve adherence to interventions {11c}

All participants will receive exactly 100 sachets to last them until their scheduled follow-up appointment. They will be asked to return any unused sachets when they come in for their follow-up to account for missed dosages. Participants will receive a daily reminder to take the prebiotic in the morning and afternoon using an online application (Auto Message) and will also be instructed to take it prior to brushing their teeth in the morning and at night as an additional reminder.

### Relevant concomitant care permitted or prohibited during the trial {11d}

All relevant concomitant care will be permitted and recorded.

### Provisions for post-trial care {30}

There will be no post trial care. It is anticipated that there will be no risks requiring compensation.

### Outcomes {12}

#### Primary outcome measures

1. Clinical assessment
  Reduction in the number of sites with PD ≥ 4 mm
  Reduction in the presence of BOP

#### Secondary outcome measures

2. Salivary markers of inflammation
  Reduction in interleukin-1ß (IL-1ß)
  Reduction in IL-6
  Reduction in CRP
  Reduction in matrix metalloproteinase-8 (MMP-8)
3. Periodontal-associated pathogens
  Reduction in *Aggregatibacter actinomycetemcomitans*
  Reduction in *Porphyromonas gingivalis*
  Reduction in *Tannerella forsythia*
  Reduction in *Treponema denticola*
4. Anthropometric parameters
  Change in BMI
5. Dietary assessment
  Change in energy intake
  Change in carbohydrate intake
  Change in fat intake
  Change in protein intake

#### Periodontal examination

All periodontal examinations will be completed by Registered Dental Hygienists with 15 years or more of experience using a periodontal probe. Multiple hygienists will participate in this study and to ensure consistency among measurements, they will be calibrated to apply 25 N of pressure by using an electronic scale [27]. Six PD sites will be measured on all teeth and implants (mesiobuccal, buccal, distobuccal, mesiolingual, lingual, and distolingual) as previously described [27]. The total number of sites with probing depths ≥ 4 mm will be measured. BOP will be measured by visual inspection and expressed as either absent or present at each of the sites.

#### Saliva collection and measurement of markers of inflammation

Participants will rinse with 85 mL of water for approximately 1 min, 10 min prior to collection. Saliva will be collected using unstimulated passive drool and a saliva collection aid (Salimetrics; Carlsbad, CA) into a prelabeled collection vial. Saliva samples will be stored on ice in a small cooler, centrifuged at 3000*g* for 15 min and supernatant collected into a labeled sterile tube and stored at − 80 °C until analysis. IL-1ß, IL-6, and CRP will be analyzed using enzyme-linked immunosorbent assay kits (Salimetrics; Carlsbad, CA) and MMP-8 will be analyzed using a human quantikine enzyme-linked immunosorbent assay kits (R&D Systems, Inc.; Oakville, Canada) as per the manufacturer’s instructions. All assays will be completed in triplicate.

#### Periodontal-associated pathogens

Participants will rinse with saline for 30 s then expectorate spit in a collection tube. This sample will be taken after the saliva sample that will be used for the measurement of markers of inflammation. Samples will be frozen at -80 °C until further analysis. *A. actinomycetemcomitans*, *P. gingivalis*, *T. forsythia*, and *T. denticola* will be quantified by quantitative PCR [28].

#### Dietary assessment

Dietary intakes will be measured using the Canadian version of the Automated Self-Administered 24-hour (ASA24®) Dietary Assessment Tool. The ASA24 will be completed online by participants and food intakes will be recalled over a 24-h period. ASA24 features two web-based applications, one that the participant will use to complete dietary recalls and one for the researcher to access nutrient analyses. Participants will be asked to complete 3 dietary assessments at each visit time point (two weekdays and one weekend day) to obtain an accurate representation. Participants will be given their username and password for the ASA24 respondent website so that they can complete the dietary assessments at home. The username will match their unique code administered on the intervention package. Participants will complete the first set of recalls before they begin the intervention. For Visits II and III, the research team will email or call the participant a week before their upcoming appointment and instruct them to log on to the ASA24 website and complete a set of food recalls before they return to the clinic for their next visit. Total energy intake, protein, fat, and carbohydrate intakes will be extrapolated from the assessments for the analysis. The means of each nutrient will be taken from the 3 recalls for each visit.

#### Anthropometric assessment

Height and weight will be measured to calculate BMI (kg/m^2^). Participants will be asked to remove their shoes and will be weighed and height will be measured to the nearest 0.1 kg and 0.1 m, respectively, using a height and weight scale (Health-O-Meter Professional; Sunbeam Products, Inc.). If certain situations prevent height and weight from being taken (COVID-19 pandemic), we will use self-reported height and weight.

### Participant timeline {13}

The participant timeline is shown in Table 2.

**Table 2:**
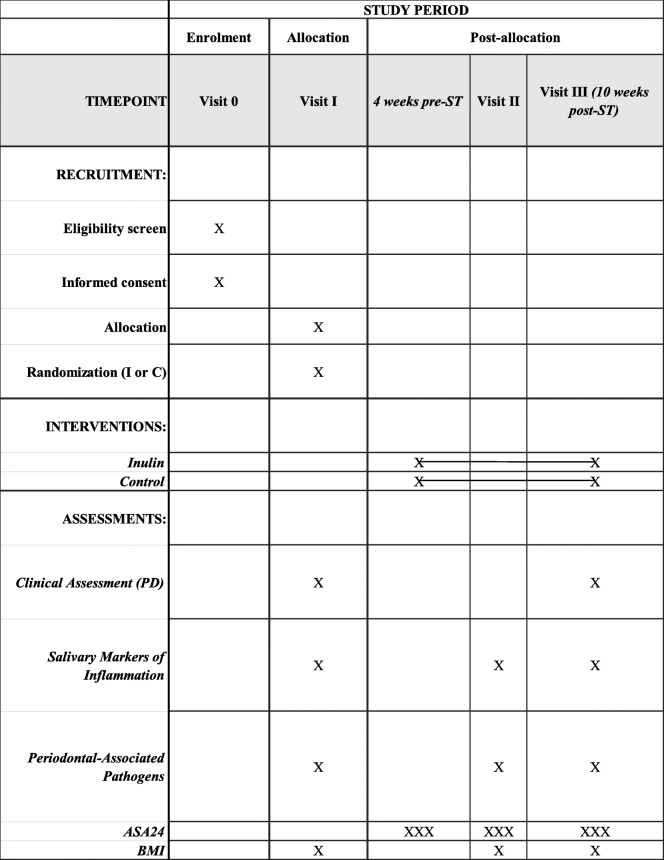
Schedule for enrollment, intervention, and assessments during the study period

### Sample size {14}

#### Sample size for pilot study

No randomized controlled trials have investigated the combined effect of inulin with ST on periodontal outcomes. To ensure the feasibility of the study—including participant adherence to the protocol and sample collection—a pilot study will be performed. The goal is to obtain data from 20 participants for each group during the pilot study. Assuming that up to 20% of participants may drop out or be withdrawn; we will aim to recruit 24 participants per group, for a total of 48 participants. If no unforeseen difficulties arise in the pilot study, participant data will be part of the full trial.

#### Sample size for full trial

The number of sites with probing depths ≥ 4 mm was used to determine sample size, as ST has been shown to reduce BOP to minimal levels to the point where an adjunct could not increase the effectiveness [3]. Previous literature has reported the mean change in number of sites with probing depths ≥ 4 mm from baseline to an average follow-up of 10.86 weeks is a mean of 81 with a standard deviation of 21 [3]. It has been shown that a high fiber diet can result in a 4% decrease in average PD though these participants were not diagnosed with periodontal disease and were not undergoing treatment with ST [24]. We believe it is reasonable to estimate that participants who are suffering from this disease and have deeper probing depths will experience a greater reduction in PD ≥ 4 mm in combination with ST (10%), as baseline PD is positively associated with follow-up PD [3]. Thus, the estimated mean change in the number of sites with probing depths of ≥ 4 mm is 91. Using a power of 80% and an alpha of 0.05, the total number of participants needed per group is 71. A 20% drop-out rate is estimated; the sample size for the full trial is 85 per group or a total of 170 participants. The sample size needed for the full trial was estimated using a statistical software (G*Power 3.1).

### Recruitment {15}

The clinic typically sees 200 patients per year for ST with 65% of the patients being nonsmokers, leaving a total of 130 patients who are eligible to participate in the study. Of those 130 individuals, 10% of people will have severe cases of periodontal disease that will require antibiotics with ST. This equates to 117 patients per year that remain eligible to participate in the study. We are estimating that 60% of people will agree to participate on the study, given the time commitment to the intervention. Previous data we collected showed 64.7% of patients undergoing periodontal surgery reported using one or more dietary supplements [29]. We anticipate that the same proportion of patients undergoing ST will be highly motivated to further support their recovery and will agree to the study. Therefore, the recruitment for 48 participants in the pilot study will be completed within 1 year. It is expected that participants from the pilot study will be included as part of the full trial sample size.

## Assignment of interventions: allocation

### Sequence generation {16a}

A computer-generated list of random numbers will be used to create a sequential set of unique codes with an equal number of participants assigned to each of the two groups.

### Concealment mechanism {16b}

A member of the research team will be responsible for the randomization and labeling of the packages. Each intervention package will contain 100 sachets of either the control or intervention product. The packages will be labeled with the unique codes that were generated. There will be a master list, kept in a locked office at the clinic, which matches the unique code with the corresponding treatment.

### Implementation {16c}

The inulin or placebo will be prepared ahead of time and stored at the clinic. The packaging will ensure that the specific treatment cannot be visually ascertained. The participants will be randomized to receive a package at the clinic and the unique code on the package will become the code that identifies the participant. Participants will be instructed to use this code to complete their dietary assessments at home. This code will also be used to label saliva samples. At this time, participants will receive verbal instructions on how to take the supplement and ask any questions they might have. Written instructions will also be included in the package to refer to when they are back home.

## Assignment of interventions: Blinding

### Who will be blinded {17a}

The trial participants and care providers will be blinded to the group allocation.

### Procedure for unblinding if needed {17b}

In the case of emergency and a participants’ allocated intervention needs to be revealed, a staff member at the clinic will match the master list to the list that identifies the participants’ name and code.

## Data collection and management

### Plans for assessment and collection of outcomes {18a}

Clinical data, including periodontal outcomes and anthropometric measurements, will be collected from electronic patient files and recorded in a specialized spreadsheet. This will be combined with data from the online dietary assessments and saliva analyses.

### Plans to promote participant retention and complete follow-up {18b}

Participants regularly attend the appointments where we will be collecting data for their clinical care. Thus, we have designed the intervention around this schedule. As previously mentioned, side effects will be minimized by taking the intervention as two separate doses during the day. Also, we have minimized the burden on the participants by having the treatments individually packaged.

### Data management {19}

Dietary questionnaires will be filled out online and will be downloaded from the ASA24 website onto a spreadsheet file that will become the main data set. Data from the clinic and saliva analyses will be added to said file upon completion and a second member of the research team will go through the imputed information to check for any errors. Regular back-ups will be made in the study folder on the protected research server/computers to prevent loss of data. Verbally recorded informed consent will be recorded in the electronic participant folder and signed paper forms will be stored at the clinic.

### Confidentiality {27}

The participant’s name and their unique code will be recorded on a separate list that will be stored in a separate location from the master list at the clinic. Once the study is completed, the list that contains the participant’s name and which unique code they received will be combined with the master list, along with clinic data including probing depth and anthropometric measurements. In order to maintain participant confidentiality, only the participant’s unique code and their corresponding data will be exported from the clinic to conduct the analyses. Saliva samples will be labeled with the participant’s unique code and the researcher conducting the analysis will not know whom they belong to. Dietary questionnaires will also be completed using the unique code.

### Plans for collection, laboratory evaluation, and storage of biological specimens for genetic or molecular analysis in this trial/future use {33}

Saliva samples will be stored at Brock University and any remaining sample will be destroyed after 10 years.

## Statistical methods

### Statistical methods for primary and secondary outcomes {20a}

Data will be entered into excel by a member of the research team. Another member will double-check the numbers and values to ensure the accuracy of data entry. Statistical analyses will be performed using IBM SPSS 26 statistical software and significance will be determined when *P* < 0.05. Mean and standard deviations will be reported for outcomes. Analysis of baseline participant demographics will be calculated to ensure consistency between the groups. The Kolmogorov-Smirnov test will be used to test the normality of the data. Levene’s test will be used to assess the equality of variances. Probing depths outcomes will be analyzed using an independent sample *t* test for the mean change in the number of PD ≥ 4 mm. If the data is not normally distributed, a Mann-Whitney test will be used. BOP is a categorical variable and chi-square analyses will be performed to measure the associations between the presence of bleeding and the treatment group.

The concentrations of the four salivary biomarkers and the quantitative values of the four periodontal pathogens at all three sample collection times will be log transformed to normalize their distribution. Two-way repeated measures analysis of variance (ANOVA) using treatment × time will be used to analyze each biomarker and pathogen individually. A repeated-measures ANOVA with Tukey’s post hoc test will be used to determine changes in energy and macronutrient intakes over the 3 visits in the intervention and control groups.

### Interim analyses {21b}

There are no interim analyses planned.

### Methods for additional analyses (e.g., subgroup analyses) {20b}

The study will also determine if there is a difference in the effect of inulin on the primary outcomes in terms of BMI. Subgroup analyses for the number of sites with PD ≥ 4 mm will be performed using two categories for BMI: normal BMI (< 25 kg/m^2^) or overweight/obese (≥ 25 kg/m^2^). This analysis will be conducted using an independent sample *t* test. Chi-square analyses will be performed for BOP.

### Methods in analysis to handle protocol non-adherence and any statistical methods to handle missing data {20c}

We want to ensure the treatment effect is not conservative; therefore a modified-to-treat approach will be used in the analysis of this trial. To be included in the analysis, participants will need to take the intervention for 75% of the 28 days pre-ST and for 75% of the 70 days post-ST. The study protocol is designed to maximize data collection. Missing data for secondary outcomes, particularly dietary intakes, the statistical method of multiple imputation will be used.

### Plans to give access to the full protocol, participant-level data, and statistical code {31c}

This study presents the full protocol. The datasets can be made available by the corresponding author when the trial concludes.

## Oversight and monitoring

### Composition of the coordinating center and trial steering committee {5d}

N/A—This is a single clinic trial involving a nutritional supplement.

### Composition of the data monitoring committee, its role and reporting structure {21a}

A data monitoring committee has not been established. This is a monocenter study design. The study results from the pilot study will be evaluated by the research team and may reveal suggestions for protocol amendments and/or a need for a data monitoring committee.

### Adverse event reporting and harms {22}

Hypersensitivity to inulin is extremely rare and mostly benign. We do not expect any adverse events to occur and participants can stop taking the intervention at any time.

### Frequency and plans for auditing trial conduct {23}

The trial will not be audited as there are no anticipated risks associated with the intervention. In addition, findings from the pilot study will be used as an opportunity to audit the trial.

### Plans for communicating important protocol amendments to relevant parties (e.g., trial participants, ethical committees) {25}

Any protocol amendments that result from the pilot or main study will first be approved by the Research Ethics Board at Brock University. Once approved, the online clinical trial registry will be updated and participants will be notified by phone or email, if applicable. To date, there have been no protocol amendments.

### Dissemination plans {31a}

We will disseminate the study findings through peer-reviewed journal publications and conference presentations. Once the manuscript has been accepted for publication, a lay summary of the study will be sent to all participants. This lay summary will include a brief statement of why the study was conducted and the aim, the salient findings, and the importance of the study including a take home message of what the study has shown. Anonymous data will be made available upon reasonable request to the corresponding author.

## Discussion

Periodontal bacteria may downregulate intestinal permeability and increase serum endotoxins or lipopolysaccharides (LPS) and subsequent systemic inflammation. In C57BL/6 mice, administration of *P. gingivalis* decreased the expression of tight junction proteins (tight junction protein 1 and occluding) which was associated with an increase of serum LPS levels [11]. LPS binds to a CD14/toll-like receptor (TLR)4/MD2 complex which initiates a cascade of inflammatory events with the secretion of pro-inflammatory cytokines [30]. The gut microbiota may mediate the physiological connection between periodontal disease, obesity, and other systemic diseases. Therefore, strategies that target changes in the gut microbiota may also affect periodontal health. Specifically, the use of inulin to support the growth of beneficial gut bacteria may help alter the oral microbiota and inflammation by targeting systemic levels of various cytokines.

Given that obesity is a major risk factor for periodontal diseases [31], we will conduct subgroup analyses for the primary outcomes in which participants are categorized as either having a BMI that is classified as normal or overweight/obese. A systematic review with a meta-analysis reviewed the efficacy of ST among obese and non-obese participants [32]. The meta-analysis showed comparable outcomes in terms of in PD and clinical attachment loss between obese and non-obese subjects. However, in two of the five studies included, participants with obesity had a smaller reduction in mean PD from baseline to follow-up. The other three studies showed comparable clinical periodontal condition in both obese and non-obese patients. The authors could not conclude if ST had a different effect in obese compared to non-obese participants with chronic periodontitis because of these conflicting results; however, they did report that serum IL-6, tumor necrosis factor-α, and leptin levels were more elevated in the obese compared to non-obese patients at both baseline and follow-up. This finding can be explained by the fact obesity results in a greater number of adipocytes and adipocytes that produce hormones and pro-inflammatory cytokines, including those found elevated in the studies, which relate to low-grade systemic inflammation [33]. Inulin supplementation at 10 g/day in patients with type 2 diabetes mellitus has demonstrated the ability in decreasing weight and BMI with significant decreases in serum high sensitivity-CRP, TNF-α, and LPS [34]. We anticipate that inulin may be able to attenuate obesity-related inflammation and as a result, overweight/obese participants may demonstrate greater reductions in PD and BOP than non-obese participants. This follows the hypothesis that systemic inflammation is one of the factors linking obesity to periodontal disease.

This study will contribute to a better understanding of the role of inulin, a type of prebiotic, and periodontal disease. To our knowledge, no direct intervention with prebiotic supplementation to support the effectiveness of ST for periodontal disease has been conducted. Though, there is evidence that a higher intake of dietary fiber supports periodontal health. Dietary fiber may act as a mechanical force on the dental surfaces to aid in the shedding of the periodontal-associated pathogens in the microbial biofilm [35]. We will determine if a supplement can still be effective for periodontal health, given the lack of mechanical force needed for digestion of the inulin supplement to be provided.

This unique study design allows us to measure the response of inulin prior to and after sanative therapy. We can determine if inulin can improve periodontal biomarkers alone and if inulin can be considered an adjunct to ST. Salivary biomarkers are likely to change prior to improvements in clinical outcomes, which is why we are measuring salivary biomarkers and periodontal pathogens but not clinical outcomes at Visit II. The first 4 weeks of the intervention prior to ST will be used to assess the response of inulin alone; we predict that inulin supplementation may be able to decrease salivary markers of inflammation and periodontal-associated pathogens. The remaining 10 weeks will compare ST to ST and inulin supplementation. It is hypothesized that the inulin group will have a greater reduction in the number of sites with PD ≥ 4 mm and for the percentage of sites with BOP. Further, the subgroup analyses are anticipated to show that the response is greater in participants who are overweight/obese.

This will be the first randomized controlled trial looking at the effects of prebiotics in periodontal outcomes completed in humans. The study design allows us to assess the effects on salivary biomarkers alone and in conjunction with treatment. A limitation of the study includes the time and resources required to recruit the participants needed to complete the full trial. Because the effect of ST at reducing the number of sites with periodontal disease is large, elucidating a potential benefit of combining inulin with ST requires a large sample size. However, periodontal disease is a chronic disease that requires lifelong maintenance such that even a small improvement in the efficiency of ST, resulting in better clinical outcomes, can help with long-term prognosis. Further enrichment with inulin may be able to sustain these benefits if it is able to modulate salivary biomarkers. This study will conclude (a) if inulin can facilitate a better periodontal state so that participants’ recovery from ST is enhanced and (b) if inulin intake can improve periodontal outcomes during ST.

## Trial status

The protocol (version 2) has been registered at ClinicalTrials.gov on 17 December 2020 (NCT04670133). The start date of the recruitment is June 1, 2022, and is estimated to be until December 31, 2023 (we were realistic about the study start date given the COVID-19 situation).

## Data Availability

Not applicable. The manuscript does not contain any data.

## Abbreviations

ANOVA: Analysis of variance
ASA24: Automated self-administered 24-hour
BMI: Body mass index
CRP: C-reactive protein
GI: Gastrointestinal
IL-1ß: Interleukin-1β
IL-6: Interleukin-6
LPS: Lipopolysaccharide
MMP-8: Matrix metalloproteinase-8
PD: Probing depth
ST: Sanative therapy
